# Clinician perspectives on patient consent for metagenomic next-generation sequencing of blood samples for the diagnosis of infection in clinical practice

**DOI:** 10.1099/jmm.0.002164

**Published:** 2026-05-07

**Authors:** Tom G.S. Williams, Helen Umpleby, Temitope Fisayo, Tommy Rampling, Catherine F. Houlihan

**Affiliations:** 1 London School of Hygiene and Tropical Medicine, London, UK; 2 Hospital for Tropical Diseases, University College London Hospitals NHS Foundation Trust, London, UK; 3 Rare and Imported Pathogens Laboratory, UK Health Security Agency, Porton Down, Salisbury, UK; 4 Portsmouth Hospitals University NHS Trust, Portsmouth, UK; 5 National Institute for Health and Care Research (NIHR) University College London Hospitals Biomedical Research Centre (BRC), London, UK; 6 Institute of Infection, Immunity and Transplantation, University College London, London, UK; 7 Department of Virology, University College London Hospitals NHS Foundation Trust, London, UK

**Keywords:** clinical metagenomics, consent, healthcare professionals, returning traveller

## Abstract

**Introduction.** Pathogen diagnostics based on metagenomic next-generation sequencing (mNGS) are now in clinical use. mNGS can identify unexpected pathogens or organisms of unclear significance and generate human genomic data. Given these features, it has been suggested that patients should provide specific informed consent for mNGS.

**Gap Statement.** There is limited published guidance on the appropriate form of consent for clinical infectious disease mNGS to guide clinical implementation and current practice varies.

**Aim.** To inform a pilot of mNGS for returning travellers delivered at a reference laboratory for use by specialist infection clinicians, we sought clinician perspectives on the form of consent required for mNGS and the information patients require to make an informed decision.

**Methodology.** A national survey of infection specialists provided clinicians' opinions.

**Results.** If consent for an infection screen including blood-borne virus testing had already been provided, only a minority of surveyed clinicians (22 out of 124, 18%) thought that mNGS should be discussed before it was performed on pre-existing blood samples.

**Conclusion.** Most of the UK infection clinicians surveyed did not think that mNGS of blood from returning travellers required discussion before being performed when patients had already consented for infection diagnostics to find the cause of their illness. However, clinicians felt that patients should be aware of the potential for additional testing and wanted information on mNGS to be readily available.

With the increasing availability of clinical infectious disease mNGS, engagement of non-specialist clinicians and patients is required to confirm the generalizability of these perspectives. The model of consent used for clinical infectious disease mNGS should be ethically adequate in addition to being acceptable to patients and clinicians.

## Data Summary

Anonymized survey data are available from the corresponding author, C.F.H., upon reasonable request.

## Introduction

Pathogen diagnostics based on metagenomic next-generation sequencing (mNGS) of patient samples are now in clinical use in the UK [1,2]. mNGS differs from established molecular diagnostics such as PCR, including those utilizing subsequent sequencing of amplified gene targets, by aiming to amplify and identify all genomic material within a sample [3].

Using this approach in clinical practice raises several challenges [4,7]. Some, such as interpreting the significance of an organism and integrating tests with imperfect sensitivity into diagnostic algorithms, are familiar to infection clinicians. Other challenges are novel, such as the potential to identify significant infections that patients may not have known could be detected with the test, including HIV, and the requirement for secure handling and effective removal of any human genomic data prior to analysis.

We consider specific (or explicit) consent for a test to refer to the need for a test to be discussed separately to the clinical indication for infection testing, with detailed information on the particular test provided for the patient to be considered adequately informed to provide consent. In the UK, specific consent for each separate infectious disease diagnostic test is generally not sought. Instead, patients typically consent for testing to determine the diagnosis and treatment of their suspected infection, with consent to individual tests inferred [8]. This includes testing for blood-borne viruses (BBVs) in some clinical contexts. For example, within emergency departments where opt-out BBV testing is performed, visual information is provided, without the requirement for explicit oral or written consent for testing [9]. In contrast, given the novel aspects of mNGS, it has been suggested that specific informed consent should be provided [4].

There is limited published guidance on the appropriate form of consent for clinical infectious disease mNGS, and practice varies. mNGS-specific consent is not provided prior to testing ventilated intensive care unit (ICU) patients’ respiratory samples [1]. In contrast, patients are assumed to have consented for mNGS testing of tissue or CSF samples by another service in the UK, though the appropriate form of consent is not described [10].

To inform a pilot service of mNGS for returning travellers delivered at a reference laboratory for use by specialist infection clinicians, we sought clinician perspectives on the form of consent required before performing mNGS on pre-existing blood samples. If specific consent was deemed necessary, our secondary goal was to identify the information patients should understand about mNGS to ensure that they are appropriately informed to provide consent.

## Methods

A clinician survey was promoted using the British Infection Association (BIA) and UK Health Security Agency (UKHSA) Imported Fever Service mailing lists. The survey was conducted using the SnapSurveys platform from 22 December 2024 to 22 January 2025 (Supplementary Information, available in the online Supplementary Material). Results of the clinician survey are presented as descriptive statistics, with categorical data summarized as frequencies of counts and associated percentages. Free-text responses were reviewed, and the topics raised were identified.

### Ethics statement

Clinician engagement was performed to inform a pilot service of mNGS for returning travellers for patients at UCLH. The mNGS pilot service was approved by the UKHSA Research Ethics and Governance Group (Study Reference Number NR0401). Clinicians were informed that their responses may be shared outside the project team prior to completing the survey (Supplementary Information, available in the online Supplementary Material). The National Health Service (NHS) Health Research Authority decision tool was used and confirmed that no ethical approval was required. All data were handled according to the General Data Protection Regulation.

## Results

### Clinician characteristics and prior knowledge of mNGS

One hundred and twenty-four clinicians completed the survey and were predominantly consultants or specialty trainees in infectious diseases, microbiology or virology (Table 1).

**Table 1.** Characteristics of clinician survey participants

		*N*=124
Characteristic		n (%)
Expertise		Single choice
	Consultant	90 (73)
	Specialty trainee	27 (22)
	Clinical scientist	4 (3)
	Clinical fellow	2 (2)
	Other	1 (1)
Specialty		Multiple choice
	Infectious diseases	87 (70)
	Microbiology	63 (51)
	Virology	30 (24)
	Other medical specialty	7 (6)
	Other	2 (2)

**Fig. 1.**
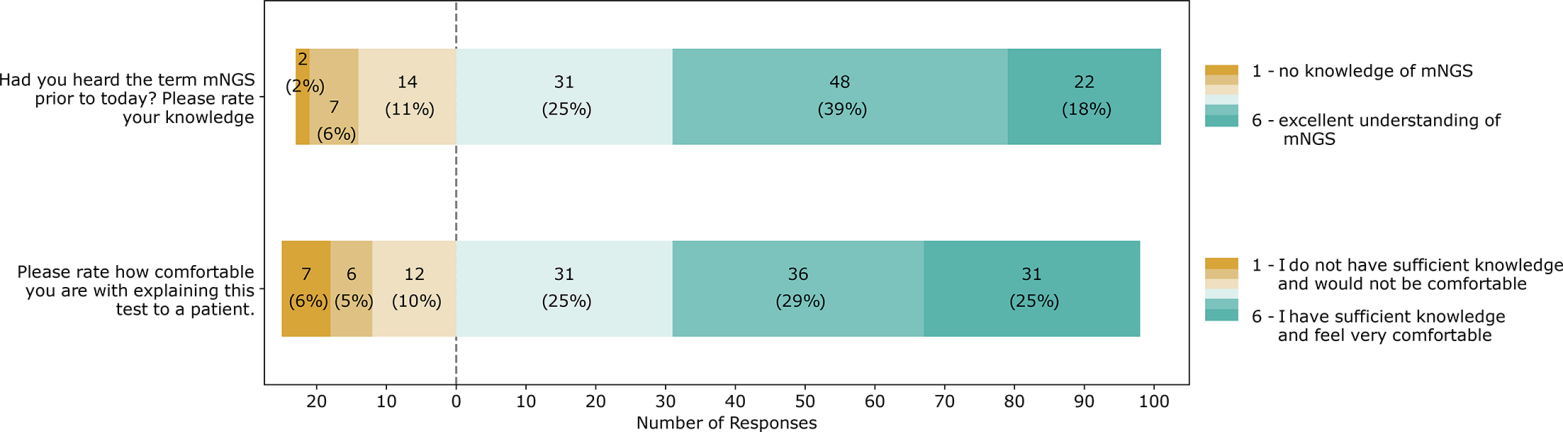
Clinician self-assessment of mNGS knowledge. Clinician survey responses using a numeric Likert scale.

Clinicians rated their knowledge on mNGS on a Likert scale from one, ‘no knowledge of mNGS’, to six, ‘excellent understanding of mNGS’. A total of 101 out of 124 (81%) selected four or higher (Fig. 1). Clinicians also rated how comfortable they were explaining this test to a patient on a Likert scale from one, ‘I do not have sufficient knowledge and would not be comfortable’, to six, ‘I have sufficient knowledge and feel very comfortable’; 98 out of 123 (80%) selected four or higher (Fig. 1). One participant did not provide a response.

The UKHSA Rare and Imported Pathogens Laboratory (RIPL) performs a panel of tests on referred samples based on the patient’s symptoms and travel history [11], rather than the referring clinician selecting pathogens to test for. A total of 114 out of 124 (92%) respondents had sent samples to RIPL from a patient they had seen clinically, and 102 of these 114 respondents (89%) do not usually inform the patient of every pathogen that will be tested for at RIPL (Table 2).

**Table 2.** Clinician survey responses

Question	Response	Single choice n (%)	Multiple choice n (%)
A returning traveller develops symptoms consistent with an acute infection. They consent to serum plus/minus other clinical samples to be sent to RIPL where serological and molecular testing for a range of pathogens will be performed. Have you sent one of these tests before (from a patient you have seen clinically)?		*N*=124	-
	Yes	114 (92)	-
	No	8 (6)	-
	Not sure	2 (2)	-
Do you usually inform the patient of every single pathogen that will be tested for?		*N*=114	-
	Yes	102 (89)	-
	No	8 (7)	-
	Not sure	4 (4)	-
Investigations at the patient’s hospital and at specialist laboratories, including RIPL, did not find a cause for their illness. Do you think a patient’s sample can undergo mNGS without informing the patient of this?		*N*=124	-
	Yes	65 (52)	-
	No	30 (24)	-
	Not sure	29 (23)	-
If the patient has agreed to blood tests for a range of infection diagnostics, including a blood-borne virus screen, do you think that mNGS can be performed without further discussion with the patient?		*N*=124	-
	Yes	86 (69)	-
	No	22 (18)	-
	Not sure	16 (13)	-
What do you feel are the important aspects for a patient to understand?		-	*N*=124
	Untargeted test that has the capability of identifying any pathogen.	-	107 (86)
	There will be human DNA in the sample, although there are steps to remove the majority of this DNA.	-	74 (60)
	Will detect HIV, hepatitis B and hepatitis C if present.	-	78 (63)
	None of these	-	4 (3)
	Other†	-	16 (13)
Ideally, should consent for mNGS be gained at the time of sample collection, or once the other planned investigations are complete and no diagnosis has been made?		*N*=124	-
	At sample collection	89 (72)	-
	Later, when no diagnosis found	26 (21)	-
	Not sure	9 (7)	-

†See main text.

### Do patients need to be informed of mNGS?

Clinicians were asked: ‘Investigations at the patient’s hospital and at specialist laboratories including RIPL did not find a cause for their illness. Do you think a patient’s sample can undergo mNGS without informing the patient of this?’ A total of 65 out of 124 (52%) responded yes, with 30 out of 124 (24%) responding ‘no’ and 29 out of 124 (23%) ‘not sure’ (Table 2).

When asked ‘If the patient has agreed to blood tests for a range of infection diagnostics including a BBV screen, do you think that mNGS can be performed without further discussion with the patient?’, the number of clinicians responding ‘yes’ increased to 86 out of 124 (69%), with 22 out of 124 (18%) responding ‘no’ and 16 out of 124 (13%) selecting ‘not sure’ (Table 2).

Clinicians were asked to explain their reasoning. Four topics were identified in their responses.

(1) Returning travellers currently provide consent to be tested for a range of pathogens, which are not individually named, to identify the cause of their illness. mNGS testing would be performed for the same purpose.

Although mNGS is being performed for the same purpose as existing consent, several respondents would adapt their practice to inform patients that mNGS was part of the testing algorithm at the point of sample collection. ‘Explicit’ consent was rarely mentioned (3 out of 124, 2%), with the potential for diagnosing HIV and the generation of human genomic data cited as reasons for this requirement.

Clinicians made comparisons with established tests. Blood cultures were highlighted as a test which can detect a range of pathogens that are not individually named when seeking consent. Some respondents considered adding 16S PCR to culture-negative samples without prior disclosure of the potential for broad molecular testing as an analogous scenario to the addition of mNGS to a negative returning traveller screen.

Some clinicians distinguished between using a validated mNGS test in a clinical setting, for which they did not feel additional consent was required, and using mNGS in research settings. Several respondents wanted an overview of mNGS test performance to be available, and one respondent felt that UK Accreditation Service accreditation was required.

(2) mNGS has the potential for incidental findings, including detecting BBVs.

The potential of mNGS to identify a pathogen unrelated to a patient’s current illness was cited as a reason to inform patients before testing. Clear reporting of incidental findings and appropriate clinical governance were highlighted as requirements by some clinicians. Responses included comparisons with incidental imaging findings, which may not have been mentioned as possibilities when seeking consent for the imaging.

Respondents mentioned that patients should be made aware when being tested for BBVs, and this also applies to mNGS if BBV testing has not already been performed. Some respondents felt that if patients had opted out of HIV testing, mNGS should not be performed.

(3) Privacy

Some respondents commented on the additional considerations required to maintain patient privacy when generating genomic data as part of a diagnostic test.

Opinions varied on whether the generation of human genomic data required mNGS to be discussed with patients. Some respondents felt that if no human genomic data were analysed, this was not required, while others felt that patients should be aware of mNGS testing regardless of how data are handled.

Several respondents wanted information on how pathogen genomic data would be stored and shared, for example, between clinical and public health teams, and whether any data would be made publicly available.

(4) Insufficient knowledge about mNGS to know if mNGS requires discussion with a patient before being performed.

Several respondents did not feel that they had sufficient understanding to provide a confident response, with technical, ethical and legal aspects of mNGS testing mentioned.

### What aspects of mNGS should patients understand?

Clinicians were asked ‘What do you feel are the important aspects for a patient to understand?’ All three options presented were identified as important by most respondents (Table 2). Other information suggested as important to communicate included the following: an overview of test performance, including sensitivity and specificity; if the test is validated; the potential for pathogens of unclear significance or no significance to the current illness to be detected; any human DNA is not analysed; and the test is newly introduced.

**Fig. 2.**
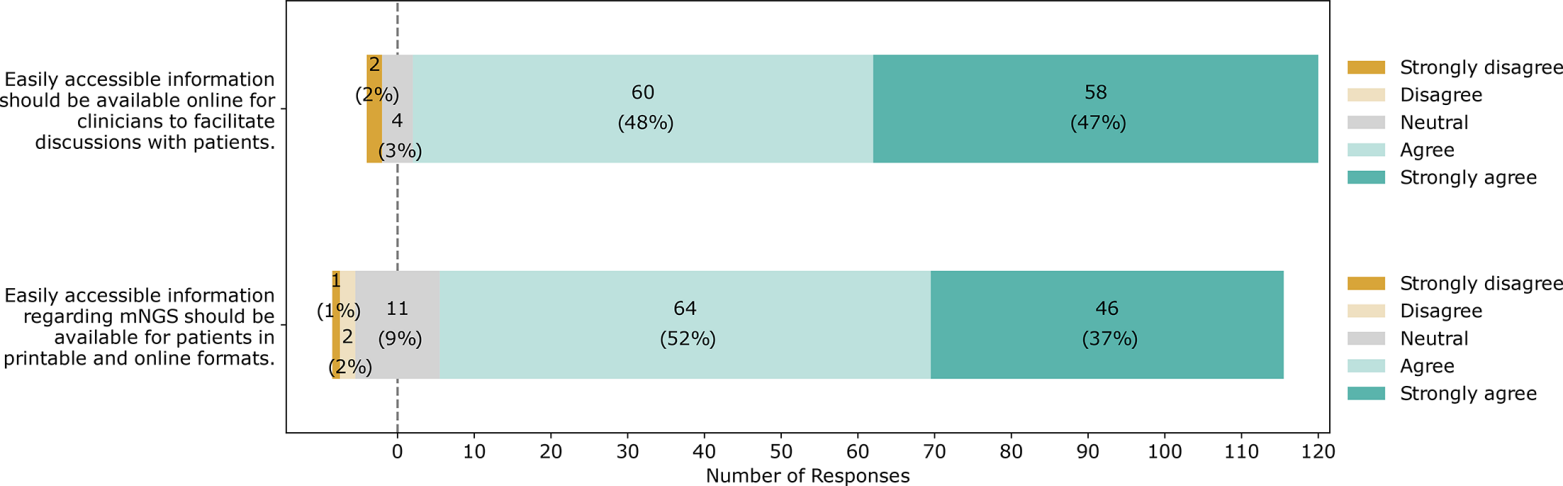
Clinician opinions on the need for accessible information on mNGS. Clinician survey responses using a Likert scale.

Most respondents felt that information about mNGS testing should be available online for clinicians to facilitate discussions with patients (Fig. 2).

## Discussion

We gathered clinician perspectives on appropriate consent for mNGS of blood samples from returning travellers to inform a pilot service delivered at a reference laboratory for use by specialist infection clinicians. Most UK infection clinicians thought mNGS did not need to be disclosed to patients prior to testing; however, around a fifth felt that mNGS should be discussed with patients, even if consent for an infection screen, including BBVs, had already been provided.

We focused on a single mNGS use case, aligning with how mNGS is transitioning to a clinical diagnostic, being operationalized on ‘a particular specimen obtained at a given time point during an infectious course’, from a ‘specific infectious syndrome and patient population’ [12].

Clinician survey participants were UK infection specialists, and most had used the existing service provided by RIPL. Although we lacked responses from clinicians in other specialties, by focusing on infection specialists, we received over a hundred responses from clinicians across the UK who were experienced in seeking consent for diagnostic testing in returning travellers. The perspectives of clinicians in non-specialist settings such as primary care and emergency departments, who may have a different level of trust in laboratory governance, were not sought and may differ substantially from infection specialists.

The reasons clinicians provided to explain their opinion on whether mNGS needed to be discussed with patients prior to testing can be placed within the analytical framework provided by the four principles of medical ethics: autonomy, beneficence, non-maleficence and justice [13]. This framework also facilitates consideration of what additional knowledge is required to reduce existing ethical uncertainties.

Most reasons given by clinicians related to their assessment of how mNGS impacted respect for patient autonomy. There was a spectrum of opinions, ranging from the view that existing consent for syndromic infection testing meant disclosure of mNGS was not required, to comments that potential secondary testing with mNGS should be disclosed for transparency as best practice, to perspectives citing concerns around incidental findings and data privacy that made mNGS discussion prior to testing essential. Clinicians considering discussion essential suggested that unexpected incidental findings from performing mNGS testing when patients were not aware of the approach could cause a breakdown in trust. This was separate to a need to respect existing decisions, such as declining BBV testing.

The generation of human genomic data, even if not analysed, was perceived by some clinicians to make mNGS disclosure essential to maintain autonomy, while others required more clarity on data handling to form an opinion. Specific consent is required for human genetic tests, where human genomic data are generated and analysed, but not for clinical samples containing human genetic material where human genomic data are not created [8]. An analogous scenario occurs with non-invasive prenatal aneuploidy testing, where actionable maternal [14] and pathogen genomic data [15] are generated but not analysed, and patient information about the test does not comment on the generation of this unanalysed genomic data [16]. Conflicting infection clinician perspectives on the ethical approach to human genomic data generated by clinical infectious disease mNGS highlight a need for clarity on data handling to support the development of a professional consensus.

Clinicians recognized that mNGS offered potential benefit to patients by increasing the probability of finding the cause of their illness, which was the original indication for infection testing, though this was balanced against the risk of a result of uncertain significance that might prompt additional and potentially unnecessary investigation. Different clinicians perceived different likelihoods for a net benefit of mNGS testing in returning travellers. This reflects the novelty of applying mNGS to this cohort as a service, rather than as research [17,20], as there is limited information for clinicians to base this judgement on. Reporting the performance of the pilot service (currently ongoing) will help to clarify the net benefit of mNGS in this patient group.

When considering whether the potential for incidental findings necessitated disclosure, clinicians also reasoned by comparison to current consent practice for established tests, including incidental findings on imaging not mentioned as possibilities in advance of testing. Such comparisons did not typically evaluate the ethical acceptability of existing practice, and, for example, it has been suggested that consent for whole-body nuclear medicine imaging should be adapted to include discussion with patients about their preference for disclosure of any findings of uncertain significance [21]. This ‘tiered’ approach to consent has been proposed for human genetic testing [22,23], where patients consent to reporting results considered to be the cause of their condition separately to reporting unsolicited significant findings or findings of uncertain significance. A ‘tiered’ approach is not currently used in clinical infection mNGS [1,2] and is also not offered by the returning traveller mNGS service.

Patient engagement through a focus group discussion and survey was also performed to inform the approach to consent taken by the returning traveller mNGS service. As patients were not informed that their responses might be shared outside the pilot project team prior to completing the survey, and not all patients could be contacted retrospectively, we have decided that it is not appropriate to publish their responses. Nonetheless, we hope that sharing our patient engagement materials (Supplementary Information, available in the online Supplementary Material) will aid other clinicians in designing patient and public involvement and engagement (PPIE) for the introduction of infection diagnostics, including both mNGS and other technologies that generate human genomic data with potential for unexpected findings such as transcriptomics. We believe that there is scope for qualitative research on this topic in a larger and more diverse patient cohort to provide generalizable findings.

Two key improvements in design would improve the interpretability of our findings. Firstly, we did not explore clinician views on appropriate consent for established diagnostic tests, which would have provided a useful comparison to their perspectives on mNGS. Secondly, presenting vignettes of alternative consent approaches to mNGS for clinicians to select between would generate more practical results. Three approaches can be discerned from the clinician perspectives we report:

1. Specific (or explicit) consent, where mNGS testing requires a separate discussion to the clinical indication for infection testing, with detailed information provided to the patient so they can be considered adequately informed to consent to mNGS.
2. Consent for the clinical indication for infection testing with disclosure of the potential for mNGS. By disclosing the potential for mNGS, this facilitates additional questions from patients seeking further information.
3. Consent for the clinical indication for infection testing, with no requirement for disclosure of the potential for mNGS.

The concept of ‘layered’ consent has been proposed for human genetic tests [23,24], where a first layer of ‘indispensable’ information is provided to all patients, with more detailed information accessible for those who seek it. This broadly corresponds to the second approach described above, where mNGS is disclosed to facilitate additional questions from patients seeking further information.

The clinician perspectives we report support an evolution of the current process of obtaining informed consent from returning travellers for infection diagnostic tests. As an example, for a patient who has returned from Brazil with a clinical picture compatible with an arboviral infection, we would suggest that consent should be sought for the clinical indication for testing with disclosure of the potential for mNGS:

‘We will send a range of tests to try and identify the infection causing your illness, including testing for viruses carried by mosquitos, like dengue. We will also test for HIV, Hepatitis B and Hepatitis C. *If initial tests are negative, additional testing to find any pathogen genetic material in your blood may be performed. No human genetic information is analysed*.’

Maintaining privacy when handling mNGS data was identified as a key issue by clinicians, for whom confidentiality is a key professional standard [24].

As clinical infectious disease mNGS diagnostics enter clinical practice, there is an opportunity for professional bodies such as the Royal College of Pathologists and government agencies such as NHS England and UKHSA to provide leadership by defining best practice on mNGS data handling. Areas which would benefit from guidance include the following: whether raw data containing human genomic data can be stored, even if this information is not analysed, or if human reads should be removed from storage after identification; the duration for which pathogen genomic data need to be retained for; and whether pathogen genomic data can be shared between hospital laboratories and public health without patient consent at the level of sequencing reads or consensus sequences. In addition, validation and accreditation processes need to ensure that clinicians can confidently inform patients that human genomic data are not analysed by mNGS tests for infection.

These areas are not covered by guidelines for validating next-generation sequencing in clinical practice focused on human genetics (e.g. [25,27]). The European Society for Clinical Virology (ESCV) Network on Next-Generation Sequencing does recommend validating human read removal prior to analysis [28]; however, different tools are available, and there is no recommended standardized approach to this validation. The ESCV recommendations do not comment on the storage of human reads in raw data [28]. Clear guidance from professional bodies on the technical aspects of mNGS data handling would support building a consensus on appropriate consent for clinical infectious disease mNGS.

Continued engagement with patients and clinicians regarding mNGS acceptability is required. Should mNGS testing for returning travellers become widely available, it will be important to include patients from across the UK in PPIE and seek the opinions of clinicians in different specialties providing care to this patient cohort.

## Supplementary material

10.1099/jmm.0.002164 Uncited Supplementary Material 1.

## Abbreviations

BBVs: blood-borne viruses
BIA: British Infection Association
CSF: cerebrospinal fluid
ESCV: European Society for Clinical Virology
HIV: human immunodeficiency virus
ICU: intensive care unit
mNGS: metagenomic next-generation sequencing
NHS: National Health Service
PPIE: patient and public involvement and engagement
RIPL: Rare and Imported Pathogens Laboratory
UCLH: University College London Hospital
UKHSA: UK Health Security Agency
