# Supplementary material for: Clinician perspectives on patient consent for metagenomic next-generation sequencing of blood samples for the diagnosis of infection in clinical practice
Source: J Med Microbiol. 2026 May 7;75(5):002164. doi: 10.1099/jmm.0.002164 (PMC13151739; doi:10.1099/jmm.0.002164)
Supplement: Uncited Supplementary Material 1. [file jmm-75-02164-s001.pdf]

Clinician perspectives on patient consent for metagenomic next generation sequencing of blood samples for the diagnosis of infection in clinical practice

Tom G.S. Williams[a,b], Helen Umpleby[c,d], Temitope Fisayo[b], Tommy Rampling[b,c,e,f], Catherine Houlihan[c,f,g]

Affiliations

a. London School of Hygiene and Tropical Medicine, London, UK
b. Hospital for Tropical Diseases, University College London Hospitals NHS Foundation Trust, London, UK
c. Rare and Imported Pathogens Laboratory, UK Health Security Agency, Porton Down, UK
d. Portsmouth Hospitals University NHS Trust, Portsmouth, UK
e. National Institute for Health and Care Research (NIHR) University College London Hospitals Biomedical Research Centre (BRC), London, United Kingdom
f. Institute of Infection, Immunity and Transplantation University College London.
g. Department of Virology, University College London Hospitals NHS Foundation Trust

Corresponding author: Dr Catherine Houlihan, Rare and Imported Pathogens Laboratory, UK Health Security Agency, Porton Down, UK; catherine.houlihan@ukhsa.gov.uk

Supplementary Information

Contents

1) Focus group discussion information 2
2) Focus group discussion structured scenario 3
3) Patient survey pre-survey information 5
4) Patient survey questions 6
5) Clinician survey pre-survey information 9
6) Clinician survey questions 11

1) Focus group discussion information

a) Pre-discussion information

The Rare and Imported Pathogens Laboratory (RIPL), part of UK Health Security Agency (UKHSA), does blood tests for lots of different bugs (microorganisms) such as dengue and zika in patients with an illness who have recently travelled abroad.

We are developing a new test – Metagenomics – which can be done on a blood sample without the need to take another blood test.

Metagenomics can be used when the patient is thought to have an infection, but the usual tests were not able to find the cause of the infection at RIPL and the patient's hospital.

Metagenomics looks at all the genetic material (e.g. DNA – this is human and other organisms' building blocks) in the blood sample and can detect <u>any</u> organism (including any virus or bacteria) if present in a high enough amount.

As researchers and doctors, we would like to explore:

1) If patients would like to be asked and agree (consent) to this test before it is done and explore the reasoning behind this.
2) Key information patients want to understand before to the test is done

b) Explanation of metagenomics provided during the discussion:

- This test will be an extra test on a blood sample when the cause for an infection has not been found.
- It can detect any microbe (bacteria/virus) if present in the blood sample.
- It does this by looking at all the DNA (genes) in the sample. From the DNA that is detected, the test can identify which microbe this DNA belongs to.
- This test can detect HIV, hepatitis B or C if present. Most patients are offered routine testing for these viruses at their hospital.
- There will be human (patient) DNA unavoidably captured in the sample as the blood test is taken. But any DNA identified as human will be removed and not looked at.
- Benefits: Can detect microbes not tested for because we do not expect them in a particular country, microbes we don't have specific tests for & new microbes.

2) Focus group discussion structured scenario

i. A patient comes to hospital with a high temperature, headache and feeling achey and tired – symptoms consistent with an infection.

   If the person has recently travelled or has a low immune system, as well as the more common infections they will also be at risk of more unusual infections.

   Tests at the hospital do not reveal a cause. A blood test is taken from the patient and sent to a specialist lab to test for a range of more unusual infections.

   The patient is told a blood test will be sent to a specialist lab for testing for a number of different microbes that can cause infections in people who have recently travelled to X or have a low immune system.

   - Do you think the patient should be informed of every microbe that will be tested?
   - Is it acceptable for the specialist lab to decide which microbes to test for based on patient's symptoms and the lab's up to date knowledge?

ii. The testing performed at the specialist lab does not find a cause for the patient's illness.

   The doctors looking after the patient and the doctors at the specialist lab think metagenomics should be performed (this can be done on the blood sample already at the specialist lab).

   - Do you think the patient needs to be asked and agree to metagenomics being performed?
   - If the patient had had or agreed to have HIV, hepatitis B & C tests done would this change your opinion?
   - What information is important for you to understand prior to the metagenomics test?
   - What format would you like this information? For example, leaflet, website link, person-person discussion

iii. A blood test sent to a specialist lab and tested for different infections can take several days to get results.

If proceeding to metagenomics was felt to be appropriate - to prevent any delay, it would be useful to know that the patient agrees to this test when the blood sample first arrives at the lab.

- Do you think metagenomics can be mentioned when the patient has the blood tests taken? E.g. "if we don't find a cause for your infection with standard tests, the specialist lab can do a new test that looks for any microbe in your blood sample". Take into account that their blood sample may never undergo metagenomics.

3) Patient survey pre-survey information

The UKHSA Rare and Imported Pathogens Laboratory (RIPL) are introducing a new test called Metagenomics.

This test is a blood test. More information on metagenomics is included below.

This survey contains examples of when the new test would be used. We would like to see whether we should be asking for patient's permission to use the test and what information patients need before the test is performed.

Metagenomics explained

Metagenomics is a special test done on a blood sample when doctors haven't found the cause of a patient's fever after doing many different tests.

This test looks at all the DNA in the blood sample. Some of the DNA will be from the patient (human DNA), this will be ignored. Only DNA from microbes (e.g. bacteria and viruses) is studied to figure out which microbe it belongs to.

This test can detect any microbe if it is present in the blood. This includes viruses such as HIV, hepatitis B and hepatitis C. Most patients will have already been tested for these viruses, during routine hospital tests.

This test can:

- Detect infections that we don’t have reliable tests for yet.
- Detect infections that are not found in certain areas of the world or don’t cause usual symptoms.
- Detect new infections that we were not previously aware of (like Covid-19).

Once the test is done, a doctor at the specialist UKHSA lab will discuss the results with an infection doctor at the hospital. If the test has identified a microbe causing the patient’s illness, the doctor will explain the results and any necessary treatment to the patient. If further testing is required to confirm the result this will also be explained.

The results of this test will take 1-2 weeks. Data is kept confidential.

What is required?

You are invited to complete the attached survey. It should only take 10 minutes to complete. You do not need any prior experience or knowledge of healthcare to complete this questionnaire.

You can complete it anonymously, there is also the option to leave your name and contact details if you are happy to be contacted to discuss this topic further.

4) Patient survey questions

Q1 THIS IS AN EXAMPLE AND NOT A MESSAGE FROM YOUR DOCTOR

You have been identified as someone who may have symptoms of infection.
If you had recently travelled abroad or have a low immune system, you could be at risk of unusual infections, as well as more common infections.

You agree to blood tests to try and find the cause of your fever. These are tested for a large range of different infections but do not find a cause for your illness.

Metagenomics is considered, would you want to be asked for your permission before this test is done? (select a single option)

- Yes
- No
- Not sure

Q2 Please briefly explain your reasoning (free text)

Q3 Some viruses that cause longstanding infections such as HIV, hepatitis B and hepatitis C would be detected if present. Testing for these viruses is routine for patients in hospital.

If you have agreed to blood tests to look for different infections including HIV, hepatitis B and hepatitis C do you think this new test can be done without further discussion with you? (select a single option)

- Yes
- No
- Not sure

Q4 If we ask patients for permission for this test, when is the best time to ask? When the blood test is taken (bear in mind that most samples won't have the test because for example, a diagnosis is reached through other tests, or the patient improves quickly and testing is no longer needed), **or** once other planned investigations have come back negative? (select a single option)

- When the blood test is done
- Later when no diagnosis has been found
- I don’t mind
- Not sure

Q5 What is the key information you would like to understand about this test? Please tick as many as apply.

- No specific information – you are in agreement for your doctors to arrange tests in your best interests to find the cause of your illness. Your doctor will explain what infections they are considering and outline the investigations, but they might not go into specific details of each individual test.
- The test will detect HIV, hepatitis B and hepatitis C if present (although may not be responsible for the current illness).
- There will be human DNA in the sample, although there are steps to remove the majority of this DNA and any remaining human DNA is not looked at.
- The test may not identify the cause of your illness. For example, if the blood test is taken when the infection is no longer in the blood (or at too low levels to be detected), or your illness is not due to an infection.
- It may take up to 2 weeks to get the result back.
- Other, please type below

Q6 How would you prefer to receive information about this test? (select a single option)

- No extra information wanted
- Doctor to patient discussion
- Leaflet
- Website link e.g. URL address or QR code
- Other, please write below

Thank you for taking the time to complete this survey. We are extremely grateful.

Demographic questions (optional)

What is your age? (select a single option)

- 18-30 years
- 31-45 years
- 46-60 years
- 61 years and over

What is your sex? (select a single option)

- Female
- Male
- Prefer not to say

Is the gender you identify with the same as your sex registered at birth? (select a single option)

- Yes
- No
- Prefer not to say

What is your ethnic group? (select a single option)

- White
- Mixed or multiple ethnic groups
- Asian or Asian British
- Black, African, Caribbean or Black British
- Other ethnic group
- Prefer not to say

Is English your first language? (select a single option)

- Yes
- No

Which ward are you currently a patient on? (select a single option)

- Infectious Diseases
- Haematology or Oncology
- Other

Did you fill this in or did someone else fill this in for you? (select a single option)

- Self
- Someone else (please document who)

5) Clinician survey pre-survey information

We have developed a metagenomics test for use in blood samples from unwell returned travellers. In order to inform the use of this test we are looking for infection clinicians to complete a short survey. The majority of questions are multiple-choice and we hope you find it interesting and enjoyable!

Introduction

The UKHSA Rare and Imported Pathogens Laboratory (RIPL) receives serum/plasma from NHS hospitals nationwide to perform tests for a range of pathogens in patients with an acute febrile illness, predominantly those with a travel history. Tests are selected depending on clinical presentation and geographical and other exposures. We have developed a metagenomic next-generation sequencing pipeline and aim to introduce this as a clinical service. Untargeted metagenomics involves sequencing and analysing all genetic material in a sample, and therefore has the capability to detect genetic material from any pathogen if present in sufficient quantities.

This will be an additional test performed on serum/plasma of patients where currently available testing has not revealed a cause for their illness, and the sample was collected at a time that it would be feasible that the pathogen's RNA/DNA would be present.

Ethical considerations

Patient DNA will unavoidably be captured in the sample. There are several steps to remove human DNA both in the laboratory and bioinformatic processes - in the latter, any sequence reads that identify as human are removed from the sequencing data and not analysed. The human reads will be in the raw sequencing data, which is on a UKHSA server and is linked to a LIMS system specimen number. Only a small group of RIPL staff can link the LIMS number to patient identifiers.

Given the untargeted nature of the test, HIV, hepatitis B and C if present would be identified. This would be fed back to the referring clinician to test locally if not known.

What is the purpose of this survey?

The aim of this survey is to help us understand:

1) Whether infection specialists deem that consent specifically for metagenomics is necessary;
2) How comfortable infection specialists are at explaining this test, what level of information they would like to know and whether clinician and/or patient leaflets would be helpful to aid discussions.
3) The most appropriate time point in a patient's journey to inform them of metagenomics.

What is required?

We estimate the survey will take less than 10 minutes to complete. No prior knowledge of metagenomics is required. Completion is anonymous although there is an option to leave your name and contact details if you would be happy to be contacted in the future for further discussion of your responses.

What will happen after you complete the survey?

A summary of findings will be written up and will be used alongside patient survey responses and public focus group discussions to determine an acceptable and practical approach to the request and consent format for metagenomics testing. The results may also be disseminated more widely.

6) Clinician survey questions

Q1 What grade are you? (select a single option)

- Consultant
- ST3 level or above
- Clinical fellow
- Other, please specify (free text)

Q2 In which speciality do you work (tick as many as applicable)?

- Infectious diseases
- Microbiology
- Virology
- Medical specialty
- Other, please specify (free text)

Q3 Had you heard the term metagenomics or metagenomic next-generation sequencing prior to today?

Please rate your knowledge (1-6). 1 = no knowledge; 6 = excellent understanding of metagenomic next-generation sequencing. (select a single option)

Q4 Please rate how comfortable you are with explaining this test to a patient.

1 = I do not have sufficient knowledge and would not be comfortable; 6 = I have sufficient knowledge and feel very comfortable. (select a single option)

Q5 A returning traveller develops symptoms consistent with an acute infection. They consent to serum plus/minus other clinical samples to be sent to RIPL where serological and molecular testing for a range of pathogens will be performed.

Have you sent one of these tests before (from a patient you have seen clinically)? (select a single option)

- Yes
- No
- Not sure

Q6 Do you usually inform the patient of every single pathogen that will be tested for? (select a single option)

- Yes
- No

- Not sure

Q7 Investigations at the patient's hospital and at specialist laboratories including RIPL did not find a cause for their illness. Do you think a patient’s sample can undergo metagenomics without informing the patient of this? (select a single option)

- Yes
- No
- Not sure

Q8 Please briefly explain your reasoning. (Free text)

Q9 If the patient has agreed to blood tests for a range of infection diagnostics including a blood-borne virus screen, do you think that metagenomics can be performed without further discussion with the patient? (select a single option)

- Yes
- No
- Not sure

Q10 What do you feel are the important aspects for a patient to understand? (Tick as many as applicable)

- Untargeted test, that has the capability of identifying any pathogen.
- There will be human DNA in the sample, although there are steps to remove the majority of this DNA.
- Will detect HIV, hepatitis B and hepatitis C if present.
- None of these
- Other, please specify (free text)

Q11 Easily accessible information should be available online for clinicians to facilitate discussions with patients. (select a single option)

- Strongly agree
- Agree
- Neutral
- Disagree
- Strongly disagree

Q12 Easily accessible information regarding metagenomics should be available for patients in printable and online formats. (select a single option)

- Strongly agree

- Agree
- Neutral
- Disagree
- Strongly disagree

Q13 Ideally, should consent for metagenomics be gained at the time of sample collection, or once the other planned investigations are complete and no diagnosis has been made? (select a single option)

- At sample collection
- Later, when no diagnosis found
- Not sure

Q14 Who completes the RIPL P1 request form at your hospital? (select a single option)

- Laboratory staff with information from clinicians
- Laboratory staff without information from clinicians
- Clinical staff not on the wards (microbiology/virology doctors)
- Clinical staff on the wards
- Not sure
- Other, please specify (Free text)

Q15 Metagenomics has the capacity to detect clinically significant and not signficant organisms. At RIPL, an MDT of clinical infection doctors, laboratory scientists and bioinformaticians will analyse the data to determine whether reads detected are significant.

Would you be satisfied with a report which states “no clinically relevant pathogens were identified” if this was the case, or would you prefer a report listing all isolates alongside a comment stating the impression of the MDT? (select a single option)

- Prefer a report detailing all isolates except laboratory contaminants with MDT comments
- Prefer a report with “no clinically significant pathogens identified”
- Prefer a report detailing all isolates including laboratory contaminants with MDT comments
- I don't know
- I don’t mind

Q16 If you have any other comments regarding the topic in this questionnaire, please write them here. (Free text)

Q17 If you are happy to be contacted to discuss your responses further, please write your name and email address below. (Free text)
